# Comparison of Chemical Compositions and Antioxidant Activities for the Immature Fruits of *Citrus changshan-huyou* Y.B. Chang and *Citrus aurantium* L.

**DOI:** 10.3390/molecules28135057

**Published:** 2023-06-28

**Authors:** Qixin Zhang, Wenying Song, Guanqi Tao, Qin Li, Lixia Wang, Wenkang Huang, Lijuan Gao, Lai Yin, Yiping Ye

**Affiliations:** 1 School of Pharmacy, Hangzhou Medical College, Hangzhou 310013, China; 2 Key Laboratory of Neuropsychiatric Drug Research of Zhejiang Province, Hangzhou Medical College, Hangzhou 311300, China; 3 Changshan Characteristic Industry Development Center, Quzhou 324000, China

**Keywords:** Aurantii Fructus, chemometric analysis, chemical comparison, *Citrus aurantium* L., *Citrus changshan-huyou* Y.B. Chang, Quzhou Aurantii Fructus

## Abstract

Quzhou Aurantii Fructus (QAF), the dried immature fruit of *Citrus changshan-huyou* Y.B. Chang, is similar to Aurantii Fructus (AF), the dried immature fruit of *Citrus aurantium* L. or its cultivars, in terms of composition, pharmacological action, and appearance. However, potential chemical markers to distinguish QAF from AF remain unknown owing to the lack of a comprehensive systematic chemical comparison aligned with discriminant analysis. To achieve a better understanding of the differences in their composition, this study aimed to identify the basic chemical compounds in QAF (*n* = 42) and AF (*n* = 8) using ultra-performance liquid chromatography coupled with electron spray ionization and quadrupole time-of-flight mass spectrometry (UPLC−QTOF/MS) and gas chromatography coupled with mass spectrometry (GC−MS). Principal component analysis (PCA), orthogonal partial least squares-discriminant analysis (OPLS−DA), and hierarchical clustering analysis (HCA) were used to further analyze, screen, and verify potential chemical markers; the antioxidant capacity was assayed in vitro. A total of 108 compounds were found in QAF and AF, including 25 flavonoids, 8 limonoids, 2 coumarins, and 73 volatile components. The chemometric analysis indicated that the main components in QAF and AF were very similar. Trace differential components, including 9 flavonoids, 2 coumarins, 5 limonoids, and 26 volatile compounds, were screened as potential chemical markers to distinguish between QAF and AF. Additionally, the antioxidant capacity of QAF was found to be greater than that of AF. This research provides insights into the quality control and clinical application of QAF.

## 1. Introduction

Rutaceae is a significant plant family that has complex kinship and contributes to food and medicine, playing an indispensable role in daily life. *Citrus changshan-huyou* Y.B. Chang (HY), a cultivar belonging to the Rutaceae family, is a hybrid of *Citrus aurantium* L. (sour orange, SC) and *Citrus grandis* (L.) Osbeck (pummelo) [1]. It has been cultivated for approximately 600 years since the Ming Dynasty, mainly in Quzhou City, Zhejiang Province; its cultivation has now become one of the mainstay industries in the local agricultural economy, offering a great variety of agricultural and subsidiary products. Quzhou Aurantii Fructus (QAF), the dried immature fruit of HY, is gathered from the end of June to July. In 2018, it was selected as the new “Zhejiang Eight Flavors” cultivar of traditional Chinese medicine, with efficacy in regulating qi and relieving stagnation. The major bioactive constituents in QAF include volatile oils, flavonoids, terpenes (especially limonoids), coumarins, and steroids and their glycosides [2,3]. QAF has been shown to possess extensive pharmacological properties, such as improving functional dyspepsia [4], antioxidant capacity [5], anti-inflammatory activity [6], anti-tussive and expectorant effects [7], hypolipidemic effect [8], hypoglycemic effect [9], protection of organs [10], and hepatoprotective activity [10,11,12].

Aurantii Fructus (AF) is the dried immature fruit of SC or its cultivars. AF has been extensively used in clinical practice owing to its excellent biological activity, hypotoxicity, and low cost. It is difficult to distinguish QAF from AF based on appearance after processing [4,13], and they have a lot similarities in pharmacological action. QAF is often used as AF in commercial products, accounting for 30% of the total market in some regions of China, because of their similar efficacy and morphological traits. However, there is a significant difference in their legal status, since AF is recorded in the “Pharmacopoeia of The People’s Republic of China” (2020 edition) (ChP), while QAF is recorded in “The Processing Standards of Traditional Chinese Medicine of Zhejiang Province” (2015 edition), limiting its application severely. Based on textual research, plant classification surveys, and comparative studies of flavonoid components and efficacy, some researchers believe that QAF is a cultivated variety of AF and that the legal status of QAF medicinally used as AF should be restored [1,14,15].

The constituents of QAF and AF have been extensively investigated individually, and previous research has shown that they have the same chemical compositions [16,17]. He et al. analyzed AF using liquid chromatography coupled with mass spectrometry (LC-MS) and gas chromatography coupled with mass spectrometry (GC−MS) and reported 104 compounds containing eight structural types [18]. Yue identified seven flavonoids and three coumarins in QAF using LC-MS, which were also found in AF [19]. The volatile compounds in QAF and AF are similar, but differences do exist [2]; however, a systematic, comprehensive comparison of the fundamental chemical constituents is still lacking, which would allow them to be distinguished.

It provides a new insight and approach to combine chromatography and chemometrics for the chemical characterization and identification of Chinese medicine. Principal component analysis (PCA), orthogonal partial least squares-discriminant analysis (OPLS−DA), and hierarchical clustering analysis (HCA) are commonly used chemometric models [20]. PCA is an unsupervised learning method that relies on dimensionality reduction and can eliminate noise and circumstantial characteristics while retaining the principal features and trends [21], which are generally used for preliminary evaluation of whether the sample has variance or whether the data have the capacity to categorize the sample [22]. OPLS−DA is a supervised discriminant analysis statistical method that uses partial least squares regression to model the relationship between data and sample categories, and OPLS−DA is mainly used to screen differential variables of samples in different groups [22,23]. HCA is a data analysis method without supervision, which creates a hierarchical nested clustering tree by calculating the similarity between different types of data points and is not affected by the expected results [24].

In this study, the volatile composition of 42 batches of QAF and 8 batches of AF was identified by GC−MS, and their flavonoids, coumarins, and limonoids compositions were detected by ultra-performance liquid chromatography coupled with quadrupole time-of-flight mass spectrometry (UPLC−QTOF/MS). Differential components were then screened and verified using chemometric analysis, including principal component analysis (PCA), orthogonal partial least squares-discriminant analysis (OPLS−DA), and hierarchical clustering analysis (HCA). Finally, the antioxidant capacity was assessed to explore the differences in the pharmacological activities of QAF and AF.

## 2. Results and Discussion

### 2.1. UPLC−QTOF/MS Analysis and Identification

**Figure 1:**
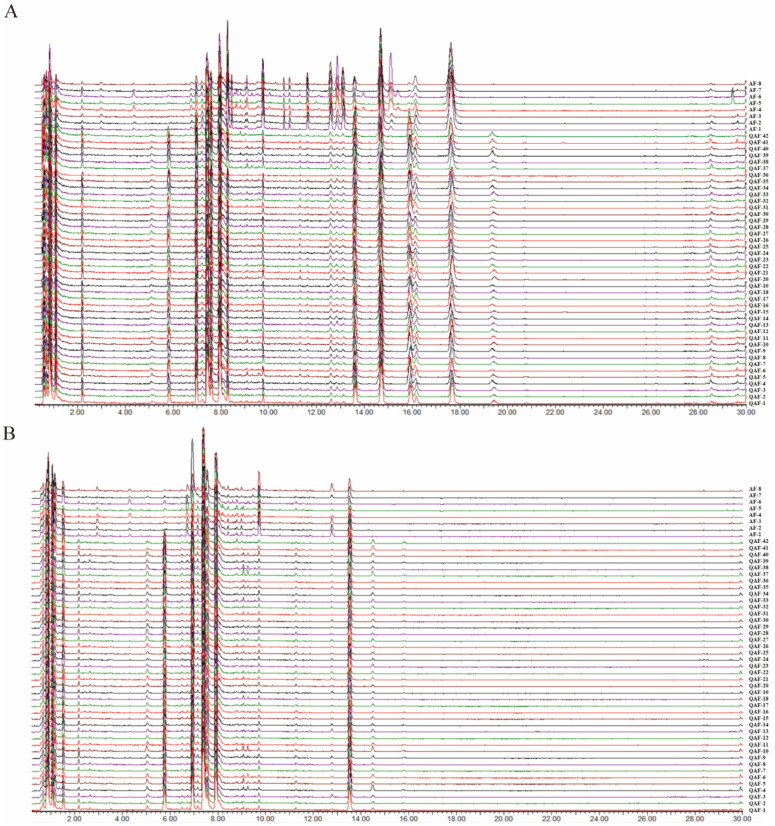
The comparison of total ion chromatograms (TIC) of UPLC−QTOF/MS of QAF and AF in positive (**A**) and negative (**B**) ions.

**Figure 2:**
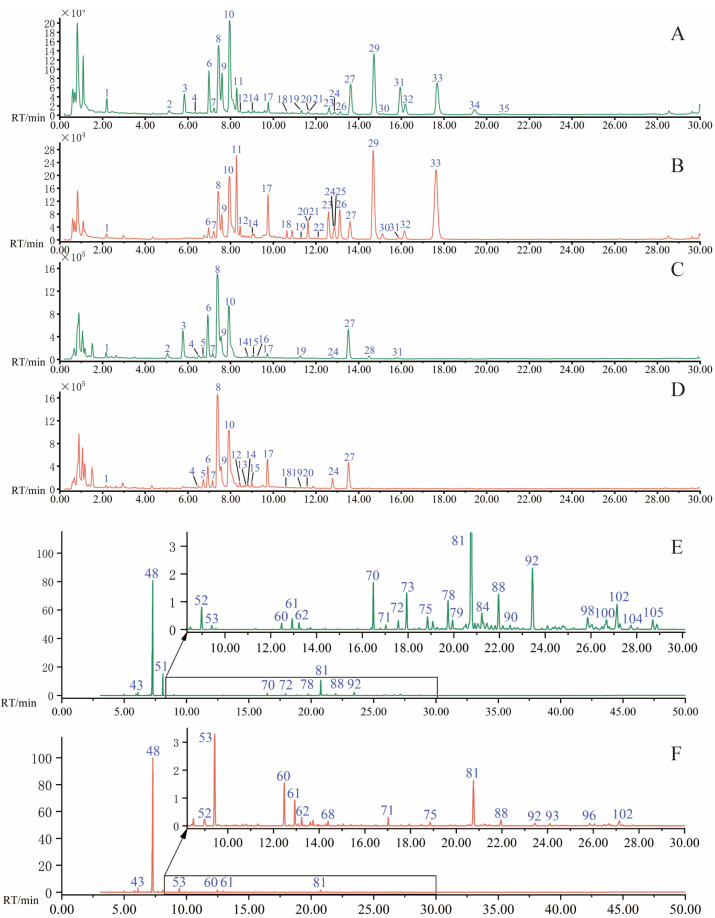
TIC of QAF and AF by UPLC−QTOF/MS under positive and negative ions and GC−MS: (**A**) TIC of QAF in positive ions; (**B**) TIC of AF in positive ions; (**C**) TIC of QAF in negative ions; (**D**) TIC of AF in negative ions; (**E**) TIC of GC−MS of QAF; and (**F**) TIC of GC−MS of AF (the range from 8.2 min to 30 min is enlarged in GC−MS).

The chemical compositions of QAF and AF were successfully identified under optimal chromatographic and MS conditions; TIC obtained in the positive and negative ion modes of 50 samples are presented in Figure 1. The major components in QAF and AF were well separated and detected within 30 min. A total of 35 compounds were identified in both QAF and AF, based on the accurate mass determination of their precursor ions and MS^E^ fragments in positive and negative ion modes. An overview of all major chromatographic peaks is presented, along with their retention times, MS data, and corresponding chemical compositions, in Table 1. The primary chemical components in QAF and AF were flavonoids, particularly flavanone, flavonoid glycosides, polymethoxyflavone, and flavone. However, other compounds were also present, such as coumarins and limonoids. The compounds are labelled in the representative chromatograms for the QAF and AF samples in positive and negative modes (Figure 2A–D). 

#### 2.1.1. Flavonoids

Flavonoids are considered the most significant bioactive constituents obtained from QAF and AF, with the same nuclear 2-phenylchromone, and are classified into flavones, flavanones, flavonols, and isoflavones. They exhibit similar dissociation mechanisms in mass spectrometry after protonation, such as the retro-Diels–Alder reaction (RDA), and absorption wavelengths in the ranges of 300–400 nm and 240–280 nm.

In this study, 25 flavonoids, mainly flavones and flavanones, were identified by UPLC-QTOF-MS. Of these, 13 compounds were analyzed and unambiguously identified by comparing their retention time, characteristic absorption wavelengths in the range 190–400 nm, and *m*/*z* values with reference standards. These compounds included naringin (**8**), neohesperidin (**10**), poncirin (**17**), naringenin (**19**), hesperetin (**20**), isosinensetin (**21**), 3′-demethylnobiletin (**22**), 6-demethoxytangeretin (**26**), nobiletin (**29**), 4′,5,6,7-tetramethoxyflavone (**30**), 3-methoxynobiletin (**32**), tangeretin (**33**), and 5-demethylnobiletin (**35**). The numbers in parentheses refer to the labels for these compounds in Figure 2A–D and Table 1.

The remaining compounds were tentatively identified by comparing the precise mass data and fragmentation patterns using the formula predictor of MassLynx and those in the literature. For instance, both compounds **2** and **3** exhibited quasimolecular ions [M + H]^+^ ions at *m*/*z* 597.1813, 597.1819 and [M − H]^−^ ions at *m*/*z* 595.1667, 595.1664, with the molecular formula C_27_H_32_O_15_. The fragment ions 289.0689, 289.0723 [M + H−C_12_H_20_O_9_]^+^ and 287.0551, 287.2336 [M – H − C_12_H_20_O_9_]^−^ were detected, indicating the release of glycone. After RDA, major fragment ions 459.8731, 459.0476 [M – H − C_8_H_8_O_2_]^−^, and 151.0897, 151.3640 [M – H − C_12_H_20_O_9_ − C_8_H_8_O_2_]^−^ for compounds **2** and **3** were obtained as a result of the damage to the original nucleus and the loss of C_8_H_8_O_2_ (136 Da); however, the presence of 417.1266, 417.1095 [M + H − C_9_H_8_O_4_]^+^ was based on the loss of C_9_H_8_O_4_ (180 Da). Compounds **2** and **3** were tentatively identified as eriocitrin and neoeriocitrin, respectively [18]. Similarly, compounds **1**, **4**, **5**, **6**, **7**, **9**, **13**, **14, 18**, and **25** were presumed to be vicenin-2, naringin 6″-rhamnoside, hesperetin 5-*O*-glucoside, narirutin, hesperetin 7-(2,6-dirhamnosylglucoside), hesperidin, naringin 6″-malonate, brutieridin, melitidin, and sinensetin, respectively, by comparing the information from previous references [18,25,26,27,28,29] and the standard database.

#### 2.1.2. Limonoids

Limonoids, highly oxygenated triterpenoid compounds, are the main cause of bitter taste in their aglycone and glucoside forms [30]. In this study, eight compounds were identified as limonoids through UPLC−QTOF/MS analysis, and their structures were either identified or provisionally assigned. Compounds **27** and **34** were identified as limonin and obacunone, respectively, based on a comparison with standard compounds. Compound **15** exhibited [M − H]^−^ ions at *m*/*z* 693.2759, inferred as quasimolecular ions with the chemical formula C_34_H_46_O_15_. Fragment ions at *m*/*z* 531.1981 [M – H − C_6_H_10_O_5_]^−^ could be generated, indicating the release of glycones. Additionally, the existence of ions at *m*/*z* 485.0921 [M – H − C_6_H_10_O_5_ − HCOOH]^−^ and *m*/*z* 161.2311 suggested the possible presence of a lactone group and the fragmentation of the chemical structure. The 487.1647 [M – H − C_6_H_10_O_5_ − CO_2_]^−^ ions were produced from decarboxylation in the seven-membered ring. Compound **15** was deduced to be a nomilin glucoside by comparison with data from a previous study [31]. Compounds **12**, **16**, **24**, **28**, and **31** were presumed to be obacunoic acid-17-*β*-D-glucoside, nomilinic acid 17-*β*-D-glucoside, obacunoic acid, nomilinic acid, and nomilin, respectively, using similar methods [18,26,27].

#### 2.1.3. Coumarins

Compounds **11** and **23** generated identical deprotonated molecule [M + H]^+^ ions at *m/z* 261.1134 and 261.1128, suggesting that they are isomers with the chemical formula C_15_H_16_O_4_. Owing to the chemical structure containing hydroxyl, carbonyl, and methoxyl groups, fragment ions at *m/z* 102.0995 [M + H − C_4_H_8_O − CH_3_O – CO − CO]^+^, 130.0093 [M + H − C_4_H_8_O − CH_3_O − CO]^+^, 158.9663 and 158.5279 [M + H − C_4_H_8_O − CH_3_O]^+^, 189.0565 and 189.0538 [M + H − C_4_H_8_O]^+^, and 243.1025 and 243.0934 [M + H − H_2_O]^+^ were found in both compounds. Compound **23** was identified as isomeranzin, based on a comparison with the reference standard. Compound **11** was deduced to be meranzin through a comparison with compound **23** and information from previous studies [18,27].

In summary, 35 common compounds were identified in both QAF and AF using UPLC−QTOF/MS, including 25 flavonoids, 8 limonoids, and 2 coumarins; among them, 4 compounds, namely, naringin 6″-rhamnoside (**4**), hesperetin 7-(2,6-dirhamnosylglucoside) (**7**), naringin 6″-malonate (**13**), and nomilin glucoside (**15**), were identified in QAF and AF for the first time.

Additionally, very similar chromatographic peaks appeared in the 42 batches of QAF from different plantings, based on both positive and negative ion modes of UPLC−QTOF/MS. These were also very similar to those in the eight batches of AF from different planting locations (Figure 1). However, there were clear distinctions in the abundance of some chromatographic peaks between QAF and AF, indicating that the quantities of the corresponding compounds differed between QAF and AF.

### 2.2. Analysis of Volatile Compounds by GC−MS

All 50 batches of samples and n-alkane solution were analyzed, and TIC was acquired in the full scan mode for QAF and AF (Figure 2C,D). 

In total, 73 compounds were identified in QAF and AF (Table 2). The relative content of the volatile compounds in QAF and AF was calculated using the area normalization method without a correction factor. The area percentages (%) of the volatile compounds in the 8 batches of AF and 42 batches of QAF are listed in Table 2. Limonene (**48**), a natural cyclic monoterpene, was the major compound in the volatile oils of both QAF and AF, with an average percentage of 65.76% (range 60.64–71.09% for 42 batches of QAF) and 85.93% (range 79.98–88.61% for 8 batches of AF), respectively. This result is consistent with those of previous studies [32,33]. The other high average percentages (≥1.00%) in QAF were *γ*-terpinene (**51**, 8.86%), germacrene D (**81**, 7.99%), germacrene B (**92**, 1.91%), *β*-myrcene (**43**, 1.13%), *α*-cadinol (**102**, 1.02%), and dysoxylonene (**88**, 1.00%); while the other high average percentages (≥1.00%) in AF were *γ*-terpinene (**51**, 2.66%), linalool (**53**, 1.86%), *β*-myrcenein (**43**, 1.62%), and germacrene D (**81**, 1.13%). Some compounds, such as sesquithujene (**74**), *γ*-elemene (**76**), *α*-guaiene (**77**), cis-*β*-farnesene (**78**), a-bulnesene (**86**), *β*-sesquiphellandrene (**89**), *δ*-cadinene (**90**), 2-(4-ethenyl-4-methyl-3-prop-1-en-2-ylcyclohexyl)propan-2-ol (**91**), (−)-globulol (**94**), guaiol (**95**), *γ*-eudesmole (**97**), isosparthulenol (**99**), cadin-4-en-10-ol (**101**), neointermedeol (**103**), isointermedeol (**104**), *β*-sinensal (**105**), juniper camphor (**106**), palmitic acid (**107**), and phytol (**108**), were only detected in QAF; the average relative content was below 0.44%. *p*-mentha-1,3,8-triene (**55**), (+)-*trans*-*p*-mentha-2,8-dien-1-ol (**56**), limonene oxide, *cis*- (**57**), (+)-*cis*-limonene 1,2-epoxide (**58**), (+)-*cis*-carveol (**67**), carvone (**68**), and perillaldehyde (**69**) were found only in AF; the average relative content was below 0.05%. These results suggest a distinct difference in the chemical composition of the volatiles present in QAF and AF.

### 2.3. Chemometric Analysis

#### 2.3.1. PCA

**Figure 3:**
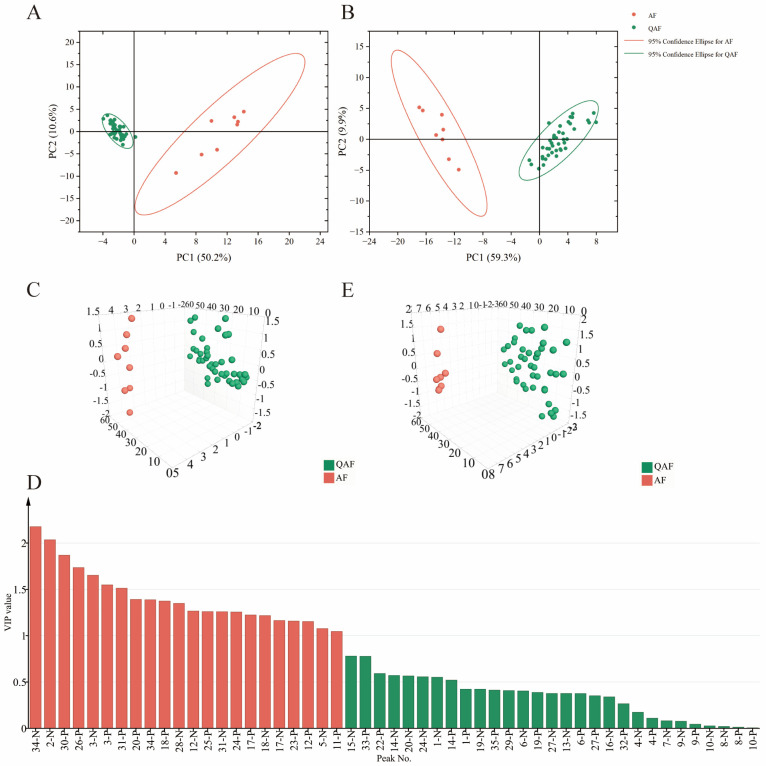
The result of chemometric analysis including: the 2D score scatterplot of PCA based on the areas of their common peaks from UPLC−QTOF/MS in the positive and negative ion modes (**A**) and GC−MS (**B**); OPLS−DA 3D score scatterplot of UPLC−QTOF/MSUPLC−QTOF/MS (**C**) and (**E**) (red and green balls represent the samples of AF and QAF, respectively); predictive VIP in OPLS−DA of UPLC−QTOF/MSUPLC−QTOF/MS (**D**) (red bars mean the compound VIP > 1).

In this study, the peak areas of 50 batches of samples from GC−MS and UPLC−QTOF/MS were set as *x* variables, and the PCA score plots were created using Origin 2023. The cumulative variance contribution of the two principal components, PC1 and PC2, accounted for 60.8% for UPLC−QTOF/MS and 69.2% for GC−MS (Figure 3A,B). In addition, the samples in each group were essentially within a 95% confidence ellipse. These results demonstrate that the PCA model was reasonable and acceptable. Two distinct groups corresponding to QAF and AF were observed based on the UPLC−QTOF/MS and GC−MS data, indicating that the chemical compounds in QAF and AF were significantly different.

As shown in Figure 3A,B, the data points for the QAF group were more closely clustered than those of the AF group, indicating that there were no clear differences in the chemical compositions in the QAF group even though these samples came from 14 different planting bases in four provinces. This was confirmed by the results of PCA based on UPLC−QTOF/MS and GC−MS data for the QAF group from 14 different planting bases (Figure S1).

#### 2.3.2. OPLS−DA

OPLS−DA is a relational model between omics data and a set of samples. In this study, a supervised OPLS−DA method was adopted to further identify differences in chemical composition between QAF and AF and to screen potential chemical markers. 

In the OPLS−DA model, Q^2^ and R^2^ are vital parameters for evaluating the rationality of the model. In this study, Q^2^ and R^2^ (Q^2^ = 0.961, R^2^ X = 0.702, R^2^ Y = 0.983) were both greater than 0.5 in UPLC−QTOF/MS according to Simca 14.1. A permutation test for OPLS−DA was performed 200 times to assess the predictability of the model (Figure S2). All test boxes were lower than the original boxes, and the intersections of curvilinear regressions and coordinate axes were in the negative semi-axis, indicating that the models were acceptable. A three-dimensional (3D) score scatterplot derived from the UPLC−QTOF/MS data is shown in Figure 3C. The data points for the 50 samples were classified into two groups corresponding to QAF and AF. These results were consistent with the results of PCA. 

To screen the differential components, variable influence on projection (VIP) was adopted. Components with a VIP value > 1 and *p* < 0.05, were selected as potential chemical markers. As shown in Figure 3D, a total of sixteen differential compounds with VIP value > 1 and *p* < 0.05 were identified; these included nine flavonoids, two coumarin and five limonoids, including eriocitrin (**2**), neoeriocitrin (**3**), hesperetin 5-*O*-glucoside (**5**), meranzin (**11**), obacunoic acid-17-*β*-D-glucoside (**12**), poncirin (**17**), melitidin (**18**), hesperetin (**20**), isomeranzin (**23**), obacunoic acid (**24**), sinensetin (**25**), 6-demethoxytangeretin (**26**), nomilinic acid (**28**), 4′,5,6,7-tetramethoxyflavone (**30**), nomilin (**31**), and obacunone (**34**). Previous research has shown that flavonoids can be used as markers to differentiate citrus varieties [34], which confirms our results.

However, the major components, including narirutin (**6**), naringin (**8**), hesperidin (**9**), neohesperidin (**10**), and naringenin (**19**), showed no difference between QAF and AF. Previous research has shown that these components were the principal biologically active ingredients assimilated in rat plasma after the oral ingestion of AF and QAF extracts [35]. The pharmacological functions of these flavonoids have been shown to mainly regulate gastrointestinal dysmotility [19], which is in accordance with the conventional clinical applications of QAF and AF. It is well-known that all the pharmacological activities of herbal medicines are significantly related to the composition of their bioactive compounds, implying that QAF and AF have very similar pharmacological effects.

The relevant R^2^ X = 0.766, R^2^ Y = 0.990, and Q^2^ = 0.981 in the OPLS−DA model from the GC−MS data indicated that the model had good prediction and goodness-of-fit. The 3D score scatterplot of OPLS−DA is displayed in Figure 3E and suggests two separate groups, corresponding to QAF and AF. Based on the permutation test results, shown in Figure S3, the models were appropriate. A total of 26 volatile compounds with VIP values > 1 and *p* < 0.05 (Figure S4) were selected as chemical markers for discriminating between QAF and AF. Previous reports have observed differences between the volatile components of QAF and AF using HS-GC-IMS [2]; however, the total average relative content of the 26 differential components in the volatile oils of QAF and AF was 13.20%.

#### 2.3.3. HCA

To further validate the results of OPLS−DA, area data of all differential peaks with VIP > 1 and *p* < 0.05 in GC−MS and UPLC−QTOF/MS were imported into Origin 2023 software for HCA.

**Figure 4:**
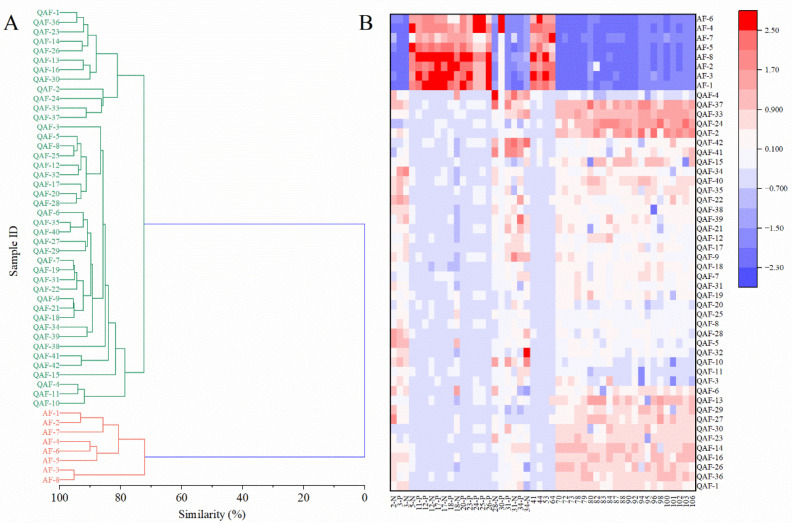
The results of HCA, including: (**A**) a cluster dendrogram (red and green samples came from AF and QAF, respectively), and (**B**) a heatmap (with the increase in peak area, the color of the bar present from blue to red, as shown in the bar at the upper right). Area data of all differential peaks with VIP > 1 and *p* < 0.05 were from UPLC−QTOF/MS in the positive and negative ion modes and GC−MS and normalized.

The data was analyzed using the group average as a clustering method and similarity as a distance type. In the cluster dendrogram, the samples were divided into two groups: one group comprised only the species QAF and the other group was composed of the species AF. Using a similarity > 70% as the standard, all 42 batches of QAF and 8 batches of AF were correctly classified by HCA (Figure 4A). The outcome of HCA was in agreement with that of the PCA and OPLS−DA, indicating that the selected differential components were valid and could discriminate between QAF and AF.

A heatmap was employed to visualize the differences between QAF and AF, which included all the peaks of the differential components (Figure 4B). The quantities of eriocitrin (**2**), neoeriocitrin (**3**), nomilinic acid (**28**), nomilin (**31**), obacunone (**34**), *δ*-elemene (**70**), copaene (**72**), (−)-cis-*β*-elemene (**73**), cis-*β*-farnesene (**78**), humulene (**79**), *γ*-muurolene (**80**), *δ*-selinene (**82**), valencen (**83**), bicyclogermacrene (**84**), *γ*-cadinene (**87**), dysoxylonene (**88**), *δ*-cadinene (**90**), germacrene B (**92**), (−)-globulol (**94**), guaiol (**95**), junenol (**96**), hinesol (**98**), T-muurolol (**100**), cadin-4-en-10-ol (**101**), *α*-cadinol (**102**), neointermedeol (**103**), and juniper camphor (**106**) were higher in QAF; the contents of the other differential components were higher in AF.

In total, all components, including nine flavonoids, two coumarins, five limonoids, and twenty-six volatile compounds, could serve as biological markers to distinguish between QAF and AF and help to verify the botanical origin of crude drugs in an application.

Rutaceae is an important source of food and medicine and has played an important role in the history of traditional Chinese medicine, mostly in qi-regulating drugs, and has a good influence on the digestive and respiratory systems on the basis of abundant flavonoids [7,36]. Flavonoids are the main components of QAF and AF that exert the pharmacological effect of regulating gastrointestinal motility [37,38], and they are also the main indicators in the comprehensive quality evaluation model of medicinal herbs [39]. As shown in the TIC (Figure 1), the main components of QAF and AF were similar in composition, but there existed differences in trace components. Chemometric analysis validated the differences presented by the TIC and presented them in a more visual way, while specific differential components were screened out, which provided a basis for the identification of herbs. Fingerprint analysis combined with clustering analysis could distinguish QAF from other Rutaceae herbs in a holistic perspective [40], but the analysis of differential components was missing. Flavonoids and volatile oils could also be used as signature components to distinguish herbs of the Rutaceae family [41,42], which was consistent with our results. In addition, the result of OPLS−DA showed that coumarins and limonoids could also be used as markers to distinguish QAF from AF.

### 2.4. Antioxidant Capacity

**Figure 5:**
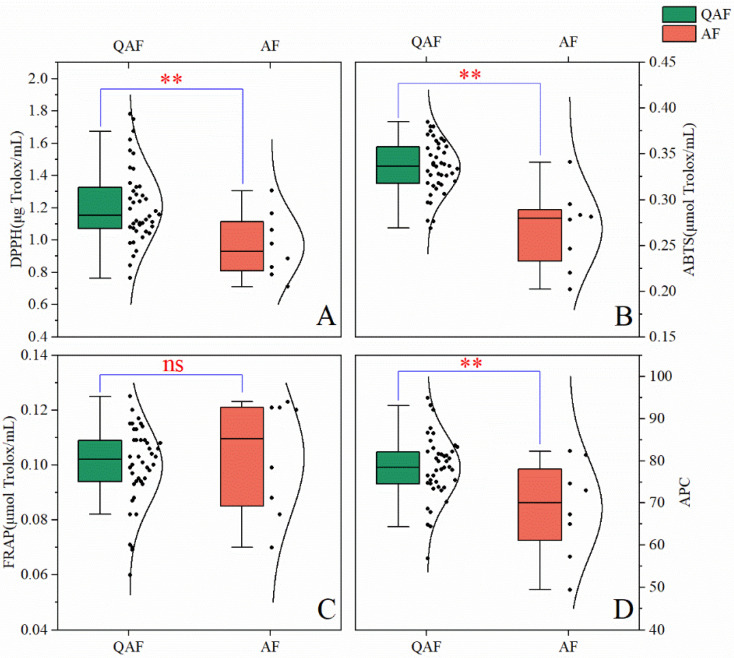
The results of antioxidant capacity: (**A**) DPPH; (**B**) ABTS; (**C**) FRAP (The Trolox equivalent antioxidant capacity was calculated for each sample, with units of μmol Trolox/mL for ABTS and FRAP and μg Trolox/mL for DPPH; a higher value indicated a stronger potency); and (**D**) APC. (*n* = 42 in QAF and *n* = 8 in AF) when AF group is compared with QAF group; ** *p* < 0.01; and ns represents *p* ˃ 0.05.

To explore the impact of the differences in composition between QAF and AF in terms of efficacy, DPPH, ABTS, and FRAP methods were used to determine the total antioxidant activity of the extract solutions of QAF and AF (Figure 5).

#### 2.4.1. Antioxidant Capability Assay

The standard curve for DPPH was *y* = 0.0137*x* + 0.0045 (R² = 0.998). The average scavenging DPPH radical for QAF and AF was 1.212 μg Trolox/mL (range 0.764–1.779 μg Trolox/mL) and 0.965 μg Trolox/mL (range 0.711–1.306 μg Trolox/mL), respectively (Figure 5A). The DPPH radical scavenging ability of QAF was significantly higher than that of AF (** *p* < 0.01).

The standard curve of ABTS was *y* = 2.6561*x* − 0.0039 (R² = 0.9988). The average scavenging ABTS radical for QAF and AF was 0.335 μmol Trolox/mL (range 0.269–0.385 μmol Trolox/mL) and 0.268 μg Trolox/mL (range 0.202–0.341 μmol Trolox/mL), respectively (Figure 5B). The ABTS radical scavenging ability of the QAF group was significantly higher than that of the AF group (** *p* < 0.01).

The ABTS standard curve was *y* = 0.1035*x* + 0.0101 (R² = 0.9985). The reducing power of QAF and AF had an average percentage of 0.100 mol Trolox/mL (range 0.060–0.125 μmol Trolox/mL) and 0.103 mol Trolox/mL (range 0.07–0.123 μmol Trolox/mL), respectively (Figure 5C). The ferric-reducing antioxidant power of QAF was similar to that of AF.

#### 2.4.2. Antioxidant Potency Composite (APC)

APC was selected to characterize the total antioxidant capacities of the samples. The APC index was computed using the method described by Seeram et al. [43]. Briefly, an identical weight coefficient was allocated to three tests, the best point in each test was set to an index value of 100, and the index points for all other samples were computed using the following equation: antioxidant index point = [(sample point/best point) × 100]. The APC values for QAF ranged from 56.81 to 94.82, while those for AF varied from 49.48 to 82.28 (Figure 5D). The APC of QAF was significantly greater than that of AF (** *p* < 0.01), suggesting that QAF has greater antioxidant ability than AF.

Oxidative stress is a predominant factor in the development of various diseases, including liver, cardiovascular, neurodegenerative, and digestive system diseases as well as psychiatric disorders, and is a potential therapeutic target [44,45,46,47,48]. Studies have shown that QAF can suppress radical production and scavenge radicals to achieve a hepatoprotective effect in vivo and in vitro [11], as well as hypolipidemic effect in hamsters with hyperlipidemia by alleviating oxidative stress [49]. The differences in antioxidant capacity between QAF and AF may impact their ability to treat certain diseases, which needs to be further verified due to different antioxidant mechanisms in vivo and in vitro. In this study, QAF and AF were compared based on their total antioxidant capacity as determined by the DPPH, ABTS, and FRAP methods. Our results suggest that the total antioxidant capacity of QAF was significantly better than that of AF, indicating that QAF has better antioxidant ability than AF, which was consistent with the previous findings [50]; however, the antioxidant capacity of QAF may change with different processing methods [51].

## 3. Materials and Methods

### 3.1. Chemicals and Reagents

All standards (purity ≥ 98.0%) were obtained from Chengdu Push Bio-technology Co., Ltd. (Sichuan, China), including naringin (PS012062), neohesperidin (PS010413), poncirin (PS010580), naringenin (PS010691), hesperetin (PS000219), isosinensetin (PS011270), 3′-demethylnobiletin (PS011281), 6-demethoxytangeretin (PS011274), limonin (PS010690), nobiletin (PS012026), 4′,5,6,7-tetramethoxyflavone (PS211213-08), 3-methoxynobiletin (PS010634), tangeretin (PS010637), obacunone (PS010281), 5-demethylnobiletin (PS011601), and meranzin (PS020754). A mixed solution of n-alkane standards was obtained from o2si Smart Solutions^®^ (North Charleston, SC, USA). Antioxidant assay kits for 1,1-Diphenyl-2-picrylhydrazyl radical (DPPH; BL897B), 2,2′-azino-bis (3-ethylbenzothiazoline-6-sulfonic acid) (ABTS; BL859B), and ferric-ion-reducing antioxidant potential (FRAP; BL858B) were purchased from Labgic Technology Co., Ltd. (Beijing, China).

### 3.2. Plant Materials

To eliminate the impact of harvest time on the constituent compounds [52,53], 42 batches of QAF and 8 batches of AF were collected from June 25th to July 11th. The AF samples were obtained from Jiangxi Province and identified as *Citrus aurantium* L. In contrast, 34 batches of QAF were harvested from 11 different planting bases in Zhejiang Province, and the remaining 8 batches of QAF were provided from Hubei (*n* = 3), Hunan (*n* = 3), and Jiangxi (*n* = 2) Provinces. All the batches were identified as *Citrus changshan-huyou* Y.B. Chang, and their voucher specimens were preserved at Hangzhou Medical College. The sample IDs, time of gathering, origin, and other pertinent information are listed in Table S1.

### 3.3. Preparation of Standard Solutions and Sample Solutions

To prepare the standard stock solutions, all the reference standards were weighed and dissolved in 50% methanol and stored at 4 °C until use.

The optimal extraction method was determined by the comparison of different methanol concentrations. The samples were crushed into a fine powder and passed through a 100 mesh (150 μm) sieve. The powder (0.25 g) was accurately weighed into a 25 mL brown glass volumetric flask with an appropriate amount of 50% methanol (*v*/*v*). The total weight was recorded, and the mixture was subjected to 20 min of ultrasonic extraction at 40 kHz. After cooling to 25 °C, the flask was reweighed and a further amount of 50% methanol (*v*/*v*) was added to offset any loss. The extracts were then filtered through 0.22 μm membranes (JLSP042201) obtained from Tianjin Keyilong Lab Equipment Co., Ltd. (Tianjin, China) and stored at 4 °C until UPLC−QTOF/MS analysis. The sample solutions for the antioxidant assay were extracted using the same method, but at different concentrations: 0.5, 1, and 16 mg of crude drug /mL for the ABTS, DPPH, and FRAP assays, respectively.

### 3.4. UPLC−QTOF/MS Analysis and Data Processing

Standard solutions and sample solutions (3 µL) were analyzed on an ACQUITY UPLC system equipped with a photodiode array (PDA) (190–400 nm) and an ACQUITY UPLC^®^ CSH^TM^ C18 column (2.1 × 100 mm, 1.7 μm) (Waters Corporation, Milford, MA, USA), maintained at 30 °C. A mobile phase comprising 0.1% aqueous formic acid solution (A) and acetonitrile (B) was used for gradient elution at a flow rate of 0.3 mL/min. The gradient program was set as follows: 0–3.5 min, 15–16% B; 3.5–5.5 min, 16–20% B; 5.5–10 min, 20–37% B; 10–20 min, 37–40% B; 20–28 min, 40–56% B; and 28–30 min, 56–100% B.

A Synapt G2 QTOF system (Waters Corporation, Milford, MA, USA) equipped with an electron spray ionization (ESI) interface was used to perform mass spectrometry under positive and negative ions in the range of *m*/*z* 100–1000 Da, using sodium formate to make the correction standard curve and leucine enkephalin as a lock mass ([M + H]^+^ 556.2771, [M − H]^−^ 554.2615) for adjustment. Nitrogen and argon were selected as the cone and collision gases, respectively. The ESI source was optimized with the following settings: 3.3/−2.5 kV capillary voltage, 120 °C source temperature, 500 L/h desolvation gas flow, 350 °C desolvation temperature, 30 V cone voltage, 50 L/h cone gas flow, 15–30 V collision energy ramp, and 20–40 V transfer collision energy ramp. Mass spectrometry data were collected for each analyte and processed in the QTOF MS^E^ mode.

Possible molecular formulae were inferred based on the parent and fragment ion information using the self-built database of compounds, previously reported compounds, and MassLynx V4.2 software (Waters Corporation, Milford, MA, USA), with a mass error of less than 5 ppm between the theoretical and measured mass values. To identify the compounds, the target compound information, key fragment ions, and fragmentation pathways were compared with those of standard compounds or those in the literature.

### 3.5. Extraction of Volatile Oil and GC−MS Analysis

Volatile oils were extracted from QAF and AF via steam distillation, according to the procedure outlined in ChP (2020 edition); dried using anhydrous sodium sulfate; and analyzed using optimal GC−MS analysis conditions after dissolving in ethyl acetate.

GC−MS analyses of the volatile oil and n-alkane solution were performed using an Agilent 7890B gas chromatograph coupled to an Agilent 5975C mass spectrometer with a triple-axis detector (TAD), equipped with an Agilent DB-5MS capillary column (30 m × 0.25 mm, 0.25 μm) and an Agilent 7693 automatic sampler (Agilent Technologies, Santa Clara, CA, USA).

The temperatures of the injector, ion source, and detector were 200 °C, 230 °C, and 270 °C, respectively. The GC oven temperature was initially held at 70 °C for 2 min, then raised to 90 °C at a rate of 5 °C/min, held for 1 min and then raised to 100 °C at 3 °C/min, then to 135 °C at 10 °C/min, then to 185 °C at a rate of 2 °C/min, held for 1 min, and finally increased to 280 °C at 20 °C/min and held for 5 min; an electron impact ionization (EI) of 70 eV was used. Data in the range of 40–400 atom mass units (amu) were collected and analyzed in SCAN mode, with a solvent delay time of 3 min.

Masshunter GC/MS acquisition B.07.06.2704 and Workflows B.08.00 (Agilent Technologies) were used for data acquisition and processing, respectively. The retention index (RI) using a DB-5MS column was calculated using closely eluted n-alkanes as the standard. The volatile compounds were identified by a comparison of the fragmentation patterns and RI with the mass spectral library in the National Institute of Standards and Technology (NIST, version NIST 17), using a standard of MS matching similarity ≥90%, and those in the literature [25,32,54].

### 3.6. Chemometric Analysis

A total of 50 samples were analyzed and the total ion chromatograms (TIC) were obtained. Automatic integration, including automatic noise measurement and smoothing, was performed for TIC of UPLC−QTOF/MS in positive and negative ions with MassLynx software to obtain the peak area of each compound. The peaks of each component in the volatile oil were also extracted by automatic integration of Workflows software to obtain a reasonable background deduction. The areas of their common peaks from UPLC−QTOF/MS in the positive and negative ion modes and GC−MS were set as *x* variables and normalized to perform chemometric analysis. PCA was used to determine whether there were differences between QAF and AF. OPLS−DA was then used to explore the potential differential components; the results were validated using HCA. Chemometric analyses were performed using Simca 14.1 (MKS Umetrics, Umea, Sweden) and Origin 2023 (OriginLab, Northampton, MA, USA).

### 3.7. Antioxidant Capacity Assays

The antioxidant capacity of the sample extract solution was determined using DPPH, ABTS, and FRAP antioxidant assay kits following the manufacturer’s instructions. The absorbance of samples and standards was measured on a BioTek Cytation 1 Cell Imaging Multimode Reader (Agilent Technologies), and data collection was performed using Gen 5 3.08 (Agilent Technologies).

Sample solutions were prepared according to the method described in Section 3.3 and diluted with 50% methanol. Appropriate concentrations were chosen to determine the absorbance within a rational scope to obtain accurate data. All results were converted to a potency at 1 mg/mL drug concentration. The Trolox equivalent antioxidant capacity was calculated for each sample, with units of μmol Trolox/mL for ABTS and FRAP and μg Trolox/mL for DPPH; a higher value indicated a stronger potency.

#### 3.7.1. DPPH Radical Scavenging Assay

DPPH was weighed and dissolved in absolute ethanol. A standard curve was generated using 0, 10, 20, 30, 40, and 60 μg/mL Trolox standard solutions, with the concentration of Trolox and the radical scavenging activity (RSA) set as the *x* and *y* variables, respectively. A 50 μL sample extract solution was mixed with 150 μL of DPPH solution in a 96-well plate; the absorbance was determined at 517 nm after being placed at 25 °C for 30 min in the dark. RSA was calculated using the following equation: RSA (%) = (1 − A/A_0_) × 100 (where A_0_ is the absorbance of the control and A is the absorbance of the sample). Finally, the antioxidant capacity was calculated using the standard curve.

#### 3.7.2. ABTS Radical Scavenging Assay

The working solution was prepared according to the manufacturer’s instructions. A standard curve was generated using 0, 0.04, 0.08, 0.16, and 0.20 μmol/mL Trolox standard solutions with the concentration of Trolox and the difference of absorbance (ΔA; ΔA = A_0_ − A, where A_0_ is the absorbance of the control and A is the absorbance of the sample) set as the *x* and *y* variables, respectively. The absorbance was measured at 419 nm after a 96-well plate containing 10 μL of sample extract solution and 190 μL of the working solution was left at 25 °C for 6 min in the dark. The ΔA value was calculated and the antioxidant capacity was determined using the standard curve.

#### 3.7.3. FRAP Assay

The working solution was prepared according to the manufacturer’s instructions. A standard curve was generated using 0, 0.4, 1.2, 2.0, 2.8, and 3.6 μmol/mL Trolox standard solutions, with the concentration of Trolox and the difference of absorbance at 590 nm set as the *x* and *y* variables, respectively. To assess the antioxidant capacity, 5 μL of sample extract solution, 25 μL of distilled water, and 170 μL of working solution were mixed in a 96-well plate at 25 °C for 10 min in the dark. The ΔA was calculated and the antioxidant capacity was determined using the standard curve.

#### 3.7.4. Statistical Analysis of Antioxidant Capacity

Three replicates were performed for each sample and data were expressed as the means ± standard deviation. Comparison between the QAF and AF groups was performed by unpaired *t*-test or Wilcoxon rank–sum test using SPSS software (version 23.0; IBM Corp., Armonk, NY, USA). Results with a *p* value *p* < 0.05 were considered statistically significant.

## 4. Conclusions

This study presents a systematic comparison of the total chemical components and antioxidant capacity of QAF and AF, using UPLC−QTOF/MS and GC−MS for the first time. A total of 108 compounds, 25 flavonoids, 8 limonoids, 2 coumarins, and 73 volatile compounds, were systemically identified as the foundational components of QAF and AF. Four of these compounds were identified in QAF and AF for the first time. The results of the chemometric analysis indicated that the main components in QAF and AF were very similar. The trace differential components, 26 volatile compounds, 9 flavonoids, 2 coumarins, and 5 limonoids, were screened as potential metabolic markers for discriminating decoctions of QAF and AF to determine their origins. Furthermore, a comparison of the total antioxidant capacity revealed that QAF had a greater antioxidant capacity than AF. As an AF cultivar, QAF can be used as a source of AF, but further investigation is required to understand its properties and applications.

These findings suggest the chemical composition characterization combined with chemometric analysis is an effective approach to distinguish the origin and determine the authenticity of Rutaceae herbs to ensure clinical efficacy and regulate the production of preparation.

## Figures and Tables

**Table 1:** The chemical compositions identified from QAF and AF by UPLC−QTOF/MS in positive and negative ion modes.

Comp No.	RT (min)	Compounds	Category	MF	Measured Mass (*m*/*z*)	Error ^b^ (ppm)	Fragment Ions (*m*/*z*)	Peak No.
1	2.16	vicenin-2	flavone	C_27_H_30_O_15_	595.1662 [M + H]^+^	−0.2	257.3252, 295.0883, 478.0288, 577.1523	1-P
					593.1505 [M − H]^−^	−0.2	162.8622, 431.2102, 473.1420, 475.1699	1-N
2	5.02	eriocitrin	flavanone	C_27_H_32_O_15_	597.1813 [M + H]^+^	−1	289.0689, 417.1266, 451.1285, 565.1464	2-P
					595.1667 [M − H]^−^	0.7	151.0897, 287.0551, 308.7538, 459.8731	2-N
3	5.76	neoeriocitrin	flavanone	C_27_H_32_O_15_	597.1819 [M + H]^+^	0	289.0723, 417.1095, 435.1271, 451.1260	3-P
					595.1664 [M − H]^−^	0.2	151.3640, 287.2366, 308.1440, 459.0476	3-N
4	6.48	naringin 6″-rhamnoside ^c^	flavanone	C_33_H_42_O_18_	727.2449 [M + H]^+^	0	151.2614, 193.0523, 342.1341, 657.7466	4-P
					725.2293 [M − H]^−^	0	273.0728, 419.1294, 511.0328, 595.1902	4-N
5	6.73	hesperetin 5-*O*-glucoside	flavanone	C_22_H_24_O_11_	463.1245 [M − H]^−^	1.1	125.0201, 217.1107, 241.2611, 374.8735	5-N
6	6.93	narirutin	flavanone	C_27_H_32_O_14_	581.1868 [M + H]^+^	−0.3	153.0183, 273.0751, 419.1333, 465.1238	6-P
					579.1712 [M − H]^−^	−0.3	151.0176, 157.7385, 271.0313, 381.9519	6-N
7	7.16	hesperetin 7-(2,6-dirhamnosylglucoside) ^c^	flavanone	C_34_H_44_O_19_	757.2553 [M + H]^+^	−0.3	303.0877, 431.1317, 449.1411, 611.2090	7-P
					755.2397 [M − H]^−^	−0.3	251.0554, 485.1661, 579.2084, 609.2543	7-N
8	7.40	naringin ^a^	flavanone	C_27_H_32_O_14_	581.1867 [M + H]^+^	−0.5	157.7210, 331.7121	8-P
					579.1713 [M − H]^−^	−0.2	273.0771, 104.1233, 419.1310, 435.1302	8-N
9	7.56	hesperidin	flavanone	C_28_H_34_O_15_	611.1973 [M + H]^+^	−0.5	303.0822, 431.1323, 465.1415, 579.1700	9-P
					609.1815 [M − H]^−^	−0.7	131.7915, 286.0830, 301.0629, 579.1698	9-N
10	7.92	neohesperidin ^a^	flavanone	C_28_H_34_O_15_	611.1976 [M + H]^+^	0	273.0708, 303.0882, 449.1455, 465.1396	10-P
					609.1819 [M − H]^−^	0	189.8311, 249.3724, 313.9281, 519.1979	10-N
11	8.25	meranzin	coumarin	C_15_H_16_O_4_	261.1134 [M + H]^+^	2.7	130.0093, 158.9663, 189.0565, 243.1025	11-P
12	8.43	obacunoic acid-17-*β*-D-glucoside	limonoid	C_29_H_32_O_17_	653.1721 [M + H]^+^	0.5	373.1664, 473.1463	12-P
					651.1559 [M − H]^−^	−0.3	263.2244, 471.1717, 583.1901, 609.1924	12-N
13	8.79	naringin 6″-malonate ^c^	flavanone	C_30_H_34_O_17_	665.1720 [M − H]^−^	0.3	271.0612, 545.2758, 501.1102, 621.1769	13-N
14	8.99	brutieridin	flavanone	C_34_H_42_O_18_	755.2411 [M + H]^+^	1.6	96.9604, 303.0997, 419.7484, 611.1989	14-P
					753.2265 [M − H]^−^	3.1	317.0820, 577.1470, 609.1931, 753.2303	14-N
15	9.07	nomilin glucoside ^c^	limonoid	C_34_H_46_O_15_	693.2759 [M − H]^−^	0.1	161.2311, 485.0921, 487.1647, 531.1981	15-N
16	9.26	nomilinic acid 17-*β*-D-glucoside	limonoid	C_34_H_48_O_16_	711.2880 [M − H]^−^	2.2	125.8538, 362.0775, 463.1354, 651.1357	16-N
17	9.73	poncirin ^a^	flavanone	C_28_H_34_O_14_	595.2027 [M + H]^+^	0	287.0934, 433.1445	17-P
					593.1868 [M − H]^−^	−0.3	177.3632, 328.8036, 428.1852, 565.3539	17-N
18	10.61	melitidin	flavone	C_33_H_40_O_17_	725.2294 [M + H]+	0.1	272.0680, 579.2518, 561.1110	18-P
					723.2137 [M − H]^−^	0.1	452.0145, 578.1447, 603.1926	18-N
19	11.27	naringenin ^a^	flavanone	C_15_H_12_O_5_	273.0768 [M + H]^+^	1.8	244.0607, 124.0807, 120.0192	19-P
					271.0606 [M − H]^−^	0	116.9294, 200.3859	19-N
20	11.58	hesperetin ^a^	flavanone	C_16_H_14_O_6_	303.0862 [M + H]^+^	−2.3	285.0684	20-P
					301.0714 [M − H]^−^	0.7	150.0010, 178.9742, 108.0344	20-N
21	11.58	iso-sinensetin ^a^	flavone	C_20_H_20_O_7_	373.1289 [M + H]^+^	0.5	163.0424, 297.1493	21-P
22	12.07	3′-demethylnobiletin ^a^	flavone	C_20_H_20_O_8_	389.1221 [M + H]^+^	−3.9	148.0940, 359.1240	22-P
23	12.54	isomeranzin ^a^	coumarin	C_15_H_16_O_4_	261.1128 [M + H]^+^	0.4	102.0995, 158.5279, 189.0538, 243.0934	23-P
24	12.79	obacunoic acid	limonoid	C_26_H_32_O_8_	473.2174 [M + H]^+^	−0.2	261.1100, 373.1358, 455.2199	24-P
					471.2008 [M − H]^−^	−2.3	137.1152, 203.5991, 391.5844, 453.1971	24-N
25	12.82	sinensetin	flavone	C_20_H_20_O_7_	373.1287 [M + H]^+^	0	312.0947, 358.1085	25-P
26	13.05	6-demethoxytangeretin ^a^	flavone	C_19_H_18_O_6_	343.118 1 [M + H]^+^	−0.3	132.0033, 218.0295, 234.5551, 282.2335	26-P
27	13.52	limonin ^a^	limonoid	C_26_H_30_O_8_	471.2019 [M + H]^+^	0	323.6053, 403.2028, 425.1961	27-P
					469.1864 [M − H]^−^	0.4	116.9230, 235.9201, 286.0120, 386.1377	27-N
28	14.49	nomilinic acid	limonoid	C_28_H_36_O_10_	531.2230 [M − H]^−^	0	126.3261, 377.4469, 471.2180, 487.2452	28-N
29	14.61	nobiletin ^a^	flavone	C_21_H_22_O_8_	403.1396 [M + H]^+^	0.7	385.0027, 311.8877, 242.0300	29-P
30	15.01	4′,5,6,7-tetramethoxyflavone ^a^	flavone	C_19_H_18_O_6_	343.1181 [M + H]^+^	−0.3	118.0903, 218.1613, 297.6151	30-P
31	15.79	nomilin	limonoid	C_28_H_34_O_9_	515.2282 [M + H]^+^	0.2	161.0510, 411.1992, 469.1833, 497.2009	31-P
					513.2133 [M − H]^−^	1.6	206.9667, 250.7199, 438.1786, 453.1919	31-N
32	16.04	3-methoxynobiletin ^a^	flavone	C_22_H_24_O_9_	433.1498 [M + H]^+^	−0.2	375.1141, 193.0924, 240.1472, 257.0406	32-P
33	17.49	tangeretin ^a^	flavone	C_20_H_20_O_7_	373.1287 [M + H]^+^	0	159.9747, 311.1900	33-P
34	19.18	obacunone ^a^	limonoid	C_26_H_30_O_7_	455.2072 [M + H]^+^	0.4	393.1996, 297.6095	34-P
					453.1915 [M − H]^−^	0.4	294.8072, 386.0809, 425.9806	34-N
35	20.55	5-demethylnobiletin ^a^	flavone	C_20_H_20_O_8_	389.1228 [M + H]^+^	−2.1	330.2824, 158.9681, 176.9824	35-P

Note: ^a^ These compounds were accurately identified with reference standards; ^b^ errors (ppm) were obtained by formula prediction software in the mass spectrometer; ^c^ these compounds were identified in QAF and AF for the first time; Arabic figures are the serial numbers of compounds according to RT; P and N represent the peaks under positive and negative ions separately in Peak No.; RT = retention time; and MF = molecular formula.

**Table 2:** The chemical compositions identified from the volatile oil of QAF and AF by GC−MS.

No.	Rt/min	Compounds	RI ^a^	MF	MW	QAF		AF	
						Average Percentage (*n* = 42)	Range	Average Percentage (*n* = 8)	Range
36	3.717	ethylbenzene	\ ^b^	C_8_H_10_	106.16	0.02%	0.01–0.02%	0.02%	0.02%
37	3.844	*p*-xylene	\	C_8_H_10_	106.16	0.10%	0.09–0.11%	0.12%	0.11–0.13%
38	4.224	*m*-xylene	\	C_8_H_10_	106.16	0.05%	0.05–0.06%	0.06%	0.05–0.06%
39	4.804	*α*-thujene	926	C_10_H_16_	136.23	0.12%	0.06–0.19%	0.07%	0.02–0.13%
40	4.985	(+)-*α*-pinene	935	C_10_H_16_	136.23	0.57%	0.35–0.83%	0.58%	0.46–0.73%
41	5.810	*β*-thujene	974	C_10_H_16_	136.23	0.05%	0.02–0.07%	0.54%	0.29–0.73%
42	5.954	*β*-pinene	981	C_10_H_16_	136.23	0.62%	0.38–0.74%	0.26%	0.11–0.74%
43	6.106	*β*-myrcene	988	C_10_H_16_	136.23	1.13%	0.95–1.35%	1.62%	1.48–1.73%
44	6.461	octanal	1003	C_8_H_10_O	128.21	0.01%	0–0.03%	0.12%	0.08–0.16%
45	6.609	*α*-phellandrene	1008	C_10_H_16_	136.23	0.05%	0.04–0.06%	0.08%	0.05–0.11%
46	6.888	*α*-terpinene	1017	C_10_H_16_	136.23	0.21%	0.18–0.24%	0.24%	0.18–0.32%
47	7.112	*o*-cymene	1025	C_10_H_14_	134.22	0.56%	0.40–0.89%	0.58%	0.04–1.13%
48	7.290	limonene	1030	C_10_H_16_	136.23	65.76%	60.64–71.09%	85.93%	79.98–88.61%
49	7.332	*β*-phellandrene	1032	C_10_H_16_	136.23	0.14%	0–0.21%	0.20%	0–0.33%
50	7.671	(*Z*)-*β*-ocimene	1043	C_10_H_16_	136.23	0.10%	0.08–0.13%	0.46%	0.38–0.62%
51	8.102	*γ*-terpinene	1057	C_10_H_16_	136.23	8.86%	7.93–9.50%	2.66%	0.47–5.98%
52	8.973	terpinolene	1085	C_10_H_16_	136.23	0.52%	0.47–0.55%	0.30%	0.21–0.53%
53	9.413	linalool	1100	C_10_H_18_O	154.25	0.10%	0.07–0.14%	1.86%	1.27–2.44%
54	9.599	nonanal	1105	C_9_H_18_O	142.24	0.02%	0–0.04%	0.02%	0–0.05%
55	9.929	*p*-mentha-1,3,8-triene	1114	C_10_H_14_	134.22	ND	ND	0.01%	0–0.04%
56	10.259	(+)-*trans*-*p*-mentha-2,8-dien-1-ol	1123	C_10_H_16_O	152.23	ND	ND	0.00%	0–0.04%
57	10.652	limonene oxide, *cis*-	1134	C_10_H_16_O	152.23	ND	ND	0.03%	0–0.04%
58	10.808	(+)-*trans*-limonene oxide	1138	C_10_H_16_O	152.23	ND	ND	0.03%	0–0.05%
59	11.312	*β*-terpineol	1152	C_10_H_18_O	154.25	0.02%	0–0.04%	0.05%	0–0.08%
60	12.470	(−)-terpinen-4-ol	1184	C_10_H_18_O	154.25	0.18%	0.13–0.23%	0.76%	0.46–1.26%
61	12.931	*α*-terpineol	1197	C_10_H_18_O	154.25	0.29%	0.19–0.40%	0.56%	0.40–0.70%
62	13.231	decanal	1207	C_10_H_20_O	156.26	0.13%	0.10–0.18%	0.15%	0.13–0.18%
63	13.324	octyl acetate	1211	C_10_H_20_O_2_	172.26	0.01%	0–0.03%	0.00%	0–0.02%
64	13.608	carveol	1222	C_10_H_16_O	152.23	0.00%	0–0.05%	0.10%	0.05–0.18%
65	13.726	nerol	1227	C_10_H_18_O	154.25	0.02%	0–0.06%	0.09%	0.03–0.18%
66	13.777	citronellol	1229	C_11_H_20_O	156.26	0.00%	0–0.02%	0.01%	0–0.04%
67	13.971	(+)-*cis*-carveol	1236	C_10_H_16_O	152.23	ND	ND	0.02%	0–0.04%
68	14.280	carvone	1248	C_10_H_14_O	150.22	ND	ND	0.05%	0.02–0.08%
69	15.045	perillaldehyde	1278	C_10_H_14_O	150.22	ND	ND	0.03%	0–0.04%
70	16.479	*δ*-elemene	1334	C_15_H_24_	204.35	0.85%	0.63–1.01%	0.03%	0–0.07%
71	17.024	neryl acetate	1355	C_12_H_20_O_2_	196.29	0.08%	0.06–0.10%	0.15%	0.10–0.20%
72	17.578	copaene	1376	C_15_H_24_	204.35	0.22%	0.15–0.27%	0.01%	0–0.05%
73	17.938	(−)-*β*-elemene	1389	C_15_H_24_	204.35	0.81%	0.63–0.98%	0.03%	0–0.08%
74	18.272	sesquithujene	1402	C_15_H_24_	204.35	0.01%	0–0.02%	ND	ND
75	18.855	caryophyllene	1420	C_15_H_24_	204.35	0.36%	0.25–0.49%	0.07%	0–0.10%
76	19.079	*γ*-elemene	1428	C_15_H_24_	204.35	0.22%	0.12–0.29%	ND	ND
77	19.274	*α*-guaiene	1434	C_15_H_24_	204.35	0.05%	0.03–0.06%	ND	ND
78	19.748	cis-*β*-farnesene	1449	C_15_H_24_	204.35	0.69%	0.47–0.90%	0.01%	0–0.04%
79	19.942	humulene	1455	C_15_H_24_	204.35	0.24%	0.17–0.32%	0.00%	0–0.03%
80	20.526	*γ*-muurolene	1474	C_15_H_24_	204.35	0.17%	0–0.35%	0.01%	0–0.04%
81	20.762	germacrene D	1481	C_15_H_24_	204.35	7.99%	5.95–9.56%	1.13%	0.83–1.52%
82	20.919	*δ*-selinene	1486	C_15_H_24_	204.35	0.13%	0.07–0.20%	0.02%	0–0.11%
83	21.050	valencen	1491	C_15_H_24_	204.35	0.20%	0.07–0.33%	0.00%	0–0.03%
84	21.223	bicyclogermacrene	1496	C_15_H_24_	204.35	0.48%	0.25–0.73%	0.02%	0–0.06%
85	21.295	*α*-muurolene	1498	C_15_H_24_	204.35	0.11%	0–0.21%	0.01%	0–0.05%
86	21.642	a-bulnesene	1508	C_15_H_24_	204.35	0.10%	0.07–0.14%	ND	ND
87	21.798	*γ*-cadinene	1512	C_15_H_24_	204.35	0.09%	0.06–0.15%	0.00%	0–0.02%
88	21.959	dysoxylonene	1517	C_15_H_24_	204.35	1.01%	0.65–1.45%	0.12%	0–0.25%
89	22.145	*β*-sesquiphellandrene	1522	C_15_H_24_	204.35	0.10%	0.07–0.14%	ND	ND
90	22.458	*δ*-cadinene	1530	C_15_H_24_	204.35	0.11%	0.06–0.19%	ND	ND
91	23.021	2-(4-ethenyl-4-methyl-3-prop-1-en-2-ylcyclohexyl)propan-2-ol	1546	C_15_H_26_O	222.37	0.03%	0–0.05%	ND	ND
92	23.439	germacrene B	1557	C_15_H_24_	204.35	1.91%	1.34–2.40%	0.05%	0–0.11%
93	24.095	spathulenol	1575	C_15_H_24_O	220.35	0.14%	0.10–0.24%	0.02%	0–0.07%
94	24.437	(−)-globulol	1584	C_15_H_26_O	222.37	0.08%	0–0.16%	ND	ND
95	24.775	guaiol	1594	C_15_H_26_O	222.37	0.20%	0.02–0.32%	ND	ND
96	25.854	junenol	1621	C_15_H_26_O	222.37	0.46%	0–0.81%	0.05%	0–0.10%
97	26.027	*γ*-eudesmole	1625	C_15_H_26_O	222.37	0.25%	0–0.49%	0.01%	0–0.12%
98	26.200	hinesol	1629	C_15_H_26_O	222.37	0.09%	0.05–0.18%	0.00%	0–0.01%
99	26.475	isosparthulenol	1636	C_15_H_24_O	220.35	0.08%	0–0.18%	ND	ND
100	26.674	t-muurolol	1641	C_15_H_26_O	222.37	0.46%	0.29–0.77%	0.02%	0–0.11%
101	26.780	cadin-4-en-10-ol	1643	C_15_H_26_O	222.37	0.08%	0–0.13%	ND	ND
102	27.135	*α*-cadinol	1652	C_15_H_26_O	222.37	1.02%	0.63–1.59%	0.15%	0–0.24%
103	27.266	neointermedeol	1655	C_15_H_26_O	222.37	0.21%	0.01–0.37%	ND	ND
104	27.748	isointermedeol	1667	C_15_H_26_O	222.37	0.10%	0–0.30%	ND	ND
105	28.700	*β*-sinensal	1690	C_15_H_22_O	218.33	0.34%	0.21–0.49%	ND	ND
106	28.877	juniper camphor	1694	C_15_H_26_O	222.37	0.19%	0.09–0.31%	ND	ND
107	40.633	palmitic acid	1965	C_16_H_32_O_2_	256.42	0.22%	0–0.71%	ND	ND
108	43.766	phytol	2105	C_20_H_40_O	296.50	0.03%	0–0.11%	ND	ND

Note: ^a^ The retention index (RI) using DB-5MS column is calculated using closely eluted n-alkanes as the standard; ^b^ the retention times of these compounds were shorter than the n-alkanes that can be detected under these conditions; and ND: the target compounds were not detected or the contents of them were less than the quantitation limit in the samples.

## Data Availability

Most of the relevant data is made available in this article. All others are provided as Supplementary Data attached to the submission.

## Supplementary Materials

The following supporting information can be downloaded at: https://www.mdpi.com/article/10.3390/molecules28135057/s1, Table S1: Sample information of Quzhou Aurantii Fructus and Aurantii Fructus. Figure S1: The PCA results of QAF from 14 different planting bases in four provinces, including: (A) UPLC−QTOF/MS PCA 2D score scatterplot; B, GC−MS PCA 2D score scatterplot (balls in blue, green, red, and black represented the sample from Zhejiang (ZJ), Jiangxi (JX), Hunan (HN), and Hubei (HB), respectively). Figure S2: OPLS−DA permutation test of UPLC−QTOF/MS for 200 times (blue and green balls represented Q^2^ and R^2^, respectively). Figure S3: OPLS−DA permutation test of GC−MS for 200 times (blue and green balls represented Q^2^ and R^2^, respectively); and (B) predictive VIP in GC−MS (red bars mean the compound VIP > 1). Figure S4: Predictive VIP in OPLS−DA of GC−MS (red bars mean the compound VIP > 1).

## List of Abbreviations

ABTS, 2,2′-azino-bis (3-ethylbenzothiazoline-6-sulfonic acid); AF, Aurantii Fructus; ChP, Pharmacopoeia of The People’s Republic of China; DPPH, 1,1-Diphenyl-2-picrylhydrazyl radical; FRAP, ferric-ion-reducing antioxidant potential; GC−MS, gas chromatography coupled with mass spectrometry; HCA, hierarchical clustering analysis; HY, *Citrus changshan-huyou* Y.B. Chang; LC-MS, liquid chromatography coupled with mass spectrometry; OPLS−DA, orthogonal partial least squares–discriminant analysis; PCA, principal component analysis; PDA, photodiode array; QAF, Quzhou Aurantii Fructus; SC, *Citrus aurantium* L.; TAD, Triple-Axis Detector; UPLC−QTOF/MS, ultra-performance liquid chromatography coupled with quadrupole time-of-flight mass spectrometry; VIP, variable influence on projection.
