# Supplementary material for: Comparison of Chemical Compositions and Antioxidant Activities for the Immature Fruits of Citrus changshan-huyou Y.B. Chang and Citrus aurantium L
Source: Molecules. 2023 Jun 28;28(13):5057. doi: 10.3390/molecules28135057 (PMC10343504; doi:10.3390/molecules28135057)
Supplement: Supplementary file 1 [file molecules-28-05057-s001.zip › molecules-2458190-supplementary.pdf]

Supplementary material

Table S1 Sample information of Quzhou Aurantii Fructus and Aurantii Fructus.

No.	Sample No.	Sample Name	Latin name	Collection time	Collection location
1	QAF-1	QAF	HY	2021/7/10	Dongxiao, Jinhua City, Zhejiang Province
2	QAF-2	QAF	HY	2021/7/10	Dongxiao, Jinhua City, Zhejiang Province
3	QAF-3	QAF	HY	2021/7/10	Dongxiao, Jinhua City, Zhejiang Province
4	QAF-4	QAF	HY	2021/7/10	Gedi Village, Changshan County, Quzhou City, Zhejiang Province
5	QAF-5	QAF	HY	2021/7/10	Gedi Village, Changshan County, Quzhou City, Zhejiang Province
6	QAF-6	QAF	HY	2021/7/10	Gedi Village, Changshan County, Quzhou City, Zhejiang Province
7	QAF-7	QAF	HY	2021/7/10	Sidu Town, Jiangshan City, Quzhou City, Zhejiang Province
8	QAF-8	QAF	HY	2021/7/10	Sidu Town, Jiangshan City, Quzhou City, Zhejiang Province
9	QAF-9	QAF	HY	2021/7/10	Sidu Town, Jiangshan City, Quzhou City, Zhejiang Province
10	QAF-10	QAF	HY	2021/7/10	Mohuan Village, Longyou County, Quzhou City, Zhejiang Province
11	QAF-11	QAF	HY	2021/7/10	Mohuan Village, Longyou County, Quzhou City, Zhejiang Province
12	QAF-12	QAF	HY	2021/7/10	Mohuan Village, Longyou County, Quzhou City, Zhejiang Province
13	QAF-13	QAF	HY	2021/7/10	Qiuchuan Town, Changshan County, Quzhou City, Zhejiang Province
14	QAF-14	QAF	HY	2021/7/10	Qiuchuan Town, Changshan County, Quzhou City, Zhejiang Province
15	QAF-15	QAF	HY	2021/7/10	Qiuchuan Town, Changshan County, Quzhou City, Zhejiang Province
16	QAF-16	QAF	HY	2021/7/10	Lianhua Town, Qujiang District, Quzhou City, Zhejiang Province
17	QAF-17	QAF	HY	2021/7/10	Lianhua Town, Qujiang District, Quzhou City, Zhejiang Province
18	QAF-18	QAF	HY	2021/7/10	Lianhua Town, Qujiang District, Quzhou City, Zhejiang Province
19	QAF-19	QAF	HY	2021/7/10	Mt.Taigong, Fanshan Village, Cangnan County, Wenzhou City, Zhejiang Province
20	QAF-20	QAF	HY	2021/7/10	Mt.Taigong, Fanshan Village, Cangnan County, Wenzhou City, Zhejiang Province
21	QAF-21	QAF	HY	2021/7/10	Mt.Taigong, Fanshan Village, Cangnan County, Wenzhou City, Zhejiang Province
22	QAF-22	QAF	HY	2021/7/10	Mt.Taigong, Fanshan Village, Cangnan County, Wenzhou City, Zhejiang Province
23	QAF-23	QAF	HY	2021/7/11	Tongyuan Town, Haiyan County, Jiaxing City, Zhejiang Province
24	QAF-24	QAF	HY	2021/7/11	Tongyuan Town, Haiyan County, Jiaxing City, Zhejiang Province

25	QAF-25	QAF	HY	2021/7/11	Tongyuan Town, Haiyan County, Jiaxing City, Zhejiang Province
26	QAF-26	QAF	HY	2021/7/11	Yongchang County, Jinhua City, Zhejiang Province
27	QAF-27	QAF	HY	2021/7/11	Yongchang County, Jinhua City, Zhejiang Province
28	QAF-28	QAF	HY	2021/7/11	Yongchang County, Jinhua City, Zhejiang Province
29	QAF-29	QAF	HY	2021/7/9	Jiantiao Town, Sanmen County, Taizhou City, Zhejiang Province
30	QAF-30	QAF	HY	2021/7/9	Jiantiao Town, Sanmen County, Taizhou City, Zhejiang Province
31	QAF-31	QAF	HY	2021/7/9	Jiantiao Town, Sanmen County, Taizhou City, Zhejiang Province
32	QAF-32	QAF	HY	2021/7/10	Tonggong Village, Changshan County, Quzhou City, Zhejiang Province
33	QAF-33	QAF	HY	2021/7/10	Tonggong Village, Changshan County, Quzhou City, Zhejiang Province
34	QAF-34	QAF	HY	2021/7/10	Tonggong Village, Changshan County, Quzhou City, Zhejiang Province
35	QAF-35	QAF	HY	2021/7/9	Wuxue City, Huanggang City, Hubie Province
36	QAF-36	QAF	HY	2021/7/9	Wuxue City, Huanggang City, Hubie Province
37	QAF-37	QAF	HY	2021/7/9	Wuxue City, Huanggang City, Hubie Province
38	QAF-38	QAF	HY	2021/7/9	Xinkai Town, Yueyang County, Yueyang City, Hunan Province
39	QAF-39	QAF	HY	2021/7/9	Xinkai Town, Yueyang County, Yueyang City, Hunan Province
40	QAF-40	QAF	HY	2021/7/9	Xinkai Town, Yueyang County, Yueyang City, Hunan Province
41	QAF-41	QAF	HY	2021/7/10	Changfu Town, Zhangshu City, Yichun City, Jiangxi Province
42	QAF-42	QAF	HY	2021/7/10	Changfu Town, Zhangshu City, Yichun City, Jiangxi Province
43	AF-1	AF	SC	2021/7/10	Nanchang City, Jiangxi Province
44	AF-2	AF	SC	2021/7/10	Nanchang City, Jiangxi Province
45	AF-3	AF	SC	2021/7/10	Nanchang City, Jiangxi Province
46	AF-4	AF	SC	2021/6/25	Changfu Town, Zhangshu City, Yichun City, Jiangxi Province
47	AF-5	AF	SC	2021/6/25	Changfu Town, Zhangshu City, Yichun City, Jiangxi Province
48	AF-6	AF	SC	2021/6/25	Changfu Town, Zhangshu City, Yichun City, Jiangxi Province
49	AF-7	AF	SC	2021/6/28	Changfu Town, Zhangshu City, Yichun City, Jiangxi Province
50	AF-8	AF	SC	2021/6/30	Changfu Town, Zhangshu City, Yichun City, Jiangxi Province

QAF and AF represented Quzhou Aurantii Fructus and Aurantii Fructus respectively, meanwhile HY and SC represented *Citrus changshan-huyou* Y.B. Chang and *Citrus aurantium* L. separately.

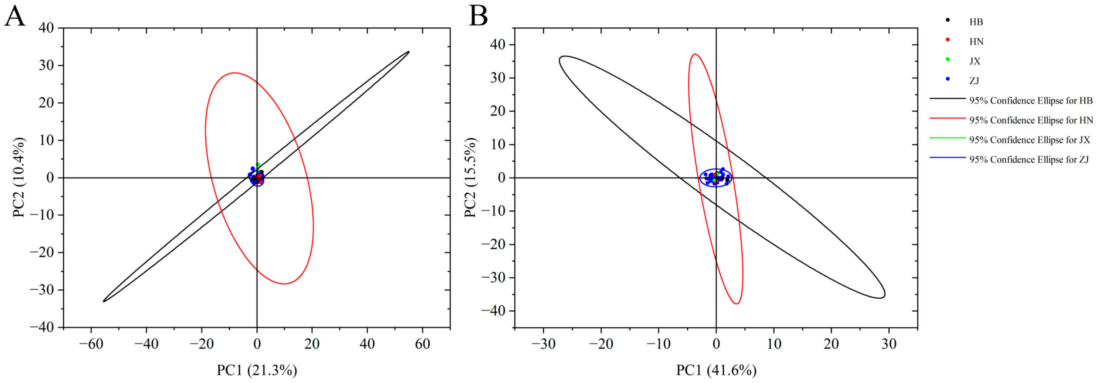

Figure S1 The PCA results of QAF from 14 different planting bases in 4 provinces, including A, UPLC-QTOF/MS PCA 2D score scatter plot; B, GC-MS PCA 2D score scatter plot (Ball in blue, green, red and black represented the sample from Zhejiang (ZJ), Jiangxi (JX), Hunan (HN) and Hubei (HB), respectively.).

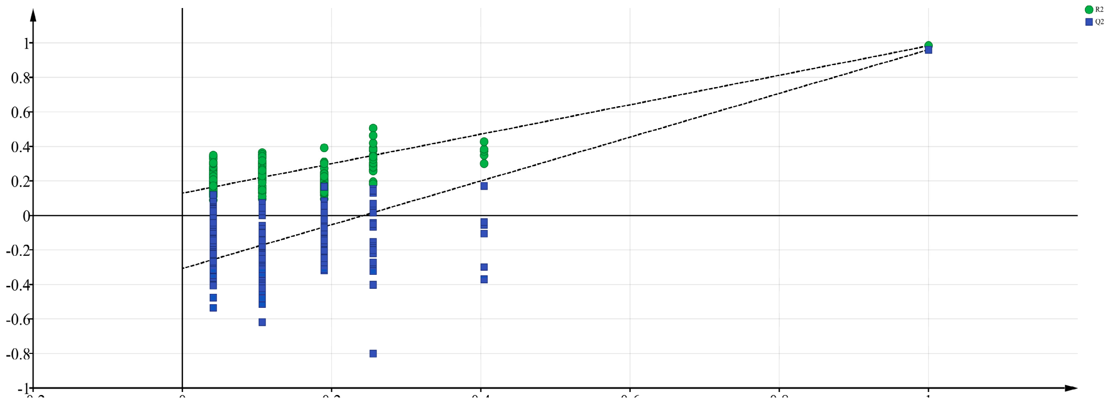

Figure S2 OPLS-DA permutation test of UPLC-QTOF/MS for 200 times (Blue and green balls represented Q^2 and R^2 respectively.).

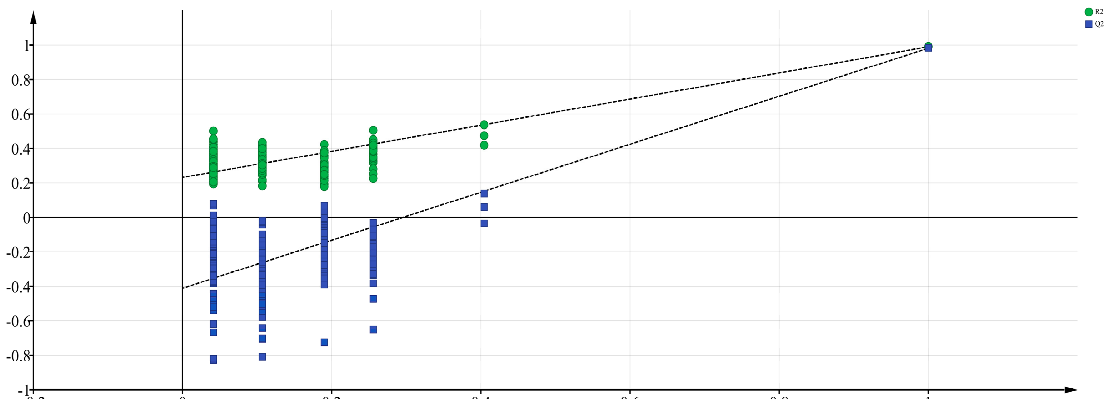

Figure S3 OPLS-DA permutation test of GC-MS for 200 times (Blue and green balls represented Q^2 and R^2 respectively.); B, predictive VIP in GC-MS (Red bars meant the compounds VIP >1.).

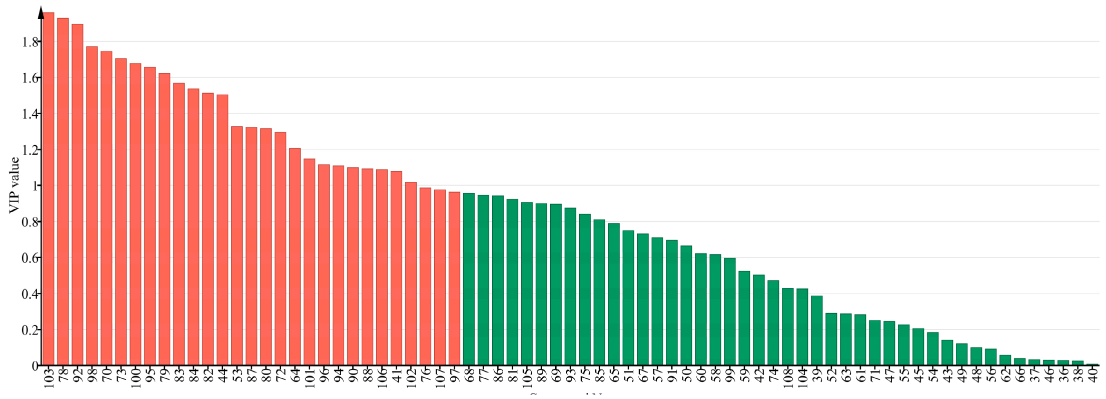

Figure S4 Predictive VIP in OPLS-DA of GC-MS (Red bars meant the compounds VIP >1.
